# Valedictory of St. Louis Probe

**Published:** 1851-03

**Authors:** 


### ARTICLE IV.

### *St. Louis Probe.*

The following is a rare specimen in the valedictory line.

*Our Journal.*—The present number closes the first volume, and ends the publication of the PROBE. During a years experience in journalism, we have been convinced that neither fame nor funds, can be acquired by conducting a medical monthly, and that many members of the medical profession are miserably poor in pocket, and more are deficient in moral principle, however well they may be imbued with the principles of their profession. We are inclined to believe that a large number, who have received our journal without paying for it, have devoted themselves to the study of scorbutus, with some success; for we must say they have treated us most scurvily, and not a few have shown a thorough acquaintance —not with abstract principles—but with the principles of abstraction, which would entitle them to the consideration of the judiciary. For the kind favors, and warm support we have received, however, from the better portion of our brethren, we return our hearty thanks, and thus take leave of them. Our hearts are so very full, and our pockets so very empty, that we are unable to say more.—*St. Louis Probe..*

